# Characterization of the global distribution and diversified plasmid reservoirs of the colistin resistance gene *mcr-9*

**DOI:** 10.1038/s41598-020-65106-w

**Published:** 2020-05-15

**Authors:** Ying Li, Xiaoyi Dai, Jing Zeng, Yan Gao, Zhikun Zhang, Luhua Zhang

**Affiliations:** 1 Department of Immunology, School of Basic Medical Sciences, Southwest Medical University, Luzhou, Sichuan China; 2 Department of Pathogenic Biology, School of Basic Medical Sciences, Southwest Medical University, Luzhou, Sichuan China

**Keywords:** Infectious diseases, Antimicrobial resistance

## Abstract

The emergence and spread of mobilized colistin resistance (*mcr*) genes have triggered extensive concerns worldwide. Here, we characterized the global distribution of *mcr-9*, a newly-identified variant of *mcr*, by assembling the data set of *mcr-9*-positive isolates from GenBank database and the literature available. Genetic features of all the *mcr-9*-harboring plasmids were determined by bioinformatic analysis. We showed that *mcr-9* is globally distributed in 21 countries across six continents, with a wide dissemination among various species of *Enterobacteriaceae* strains from human, animal, food and environment. IncHI2-ST1 plasmids were found to be the predominant replicon type carrying *mcr-9*. Comparative genomics highlighted that IncHI2-type plasmids may also serve as a critical reservoir of *mcr-9*, from which different types of circulating plasmids acquired the *mcr-9*. Results revealed that the *rcnR-rcnA-pcoE-pcoS-IS*903*-mcr-9*-*wbuC* structure was consistent in most *mcr-9* cassettes, suggesting a relatively unitary model involved in the mobilization of *mcr-9*. It is most likely that the spread of *mcr-9* was mainly attributed to the conjugation and recombination events of *mcr-9*-carrying plasmids. In summary, our results provide a comprehensive picture of the distribution and genetic environment of *mcr-9*, and demonstrate the central roles played by IncHI2 plasmids in the worldwide dissemination of *mcr-9*.

## Introduction

Antibiotic resistance poses a great threat to global public health and carbapenem-resistant *Enterobacteriaceae* is triggering a health crisis worldwide^1,2^. Colistin, a cationic cyclic-peptide, is one of the last-resort antibiotics to defend against severe infections caused by carbapenem-resistant *Enterobacteriaceae*^3^. However, since the initial discovery of a plasmid-mediated mobilized colistin resistance gene (*mcr-1*) in China in late 2015^4^, a number of diversified bacterial strains carrying *mcr-1* have been detected across over 50 countries covering six continents^5^. The prevalent plasmid-borne MCR enzyme can catalyze chemical addition of phosphoethanolamine to lipid A moiety of bacterial lipopolysaccharides, the target of colistin, which consequently promotes colistin resistance^6^.

In recent years, a growing number of *mcr*-like genes (namely, from *mcr-2* to *mcr-10*) have been identified^7–14^. These ongoing discoveries indicate a rapid evolution of MCR family under selective pressures, which raise global health concerns. *mcr-9* is a newly emerging variant of the mobilized colistin resistance determinants, which was first identified in a clinical *Salmonella enterica* isolate in the USA in May, 2019^13^. Since its initial identification, *mcr-9* has been reported in several other countries, such as China^15^, Sweden^16^, and France^17^. Not only that, *in silico* analysis using the GenBank database indicated that *mcr-9* had already been presented in a number of *Enterobacteriaceae* isolates recovered worldwide^13,17^. The high prevalence of *mcr-9* suggests one more threat to public health. However, little information is available about the global epidemiology and dissemination patterns of *mcr-9*.

To explore these issues, here we studied the geographic and host distribution of *mcr-9*-carrying strains, and investigated the genomic features of various types of *mcr-9*-harboring plasmids from an extensive collection of publicly available sequence data sourced from the NCBI repository by bioinformatics analysis. Our findings may contribute to a better understanding of the prevalence and dissemination of the newly identified *mcr-9* gene, and be helpful for developing better strategies to manage its spread.

## Results and discussion

### Geographic and isolation source of *mcr-9*

**Figure 1:**
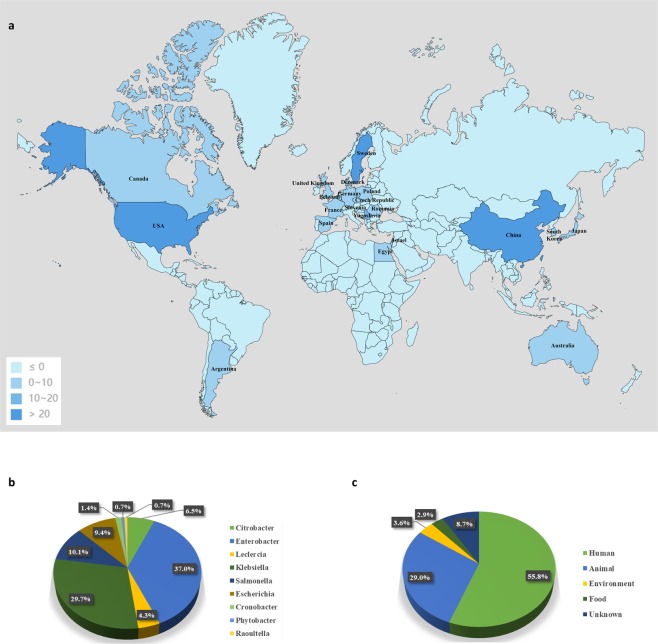
Overview of the distribution of *mcr-9*. (**a)** Worldwide distribution of *mcr-9*-positive isolates. The map was generated using the online software dituhui (https://g.dituhui.com/). **(b)** Distribution of host species harboring *mcr-9*. **(c)** Distribution of the isolation source of *mcr-9*-positive strains.

To obtain a comprehensive picture of the distribution of *mcr-9*, we blasted for *mcr-9* in the NCBI GenBank database (as of 12th February 2020, n = 78) using a 99% identity cutoff and retrieved 71 complete plasmids and 6 chromosomes containing a full-length hit to *mcr-9*. A systematic review of the literature on *mcr-9* published until 12 February 2020 resulted in the inclusion of 6 articles^13,15–19^. As a result, a total of 138 *mcr-9*-harboring strains were included for statistics. The statistical results showed that isolates carrying *mcr-9* were identified in 21 countries across six continents (Fig. 1a), including Europe (n = 72; 52.2%), Asia (n = 27; 19.6%), North-America (n = 27; 19.6%), Oceania (n = 10; 7.2%), South-America (n = 1; 0.7%) and Africa (n = 1; 0.7%). The large number of *mcr-*9-positive isolates from Europe was notable, which can be largely ascribed to the inclusion of 30 isolates from horses in Sweden^16^ and 28 from human in Europe by a previous clinical surveillance program^18^. Anyway, the high incidence of *mcr-9* in Europe is alarming, which demands more attention. In addition, the prevalence of *mcr-9* around the world might be underestimated, due to the limited availability of its epidemiological investigations. Large-scale surveillance and molecular epidemiological studies are urgently required to better understand the global distribution and dissemination of *mcr-9*, thereby facilitating establishment of effective treatments to control its spread.

According to the current data, various kinds of *Enterobacteriaceae* isolates were found to disseminate *mcr-9*, among which *Enterobacter* spp. were the most common ones (n = 51, 37.0%), followed by *Klebsiella* spp. (n = 41, 30.0%), *Salmonella* spp. (n = 14, 10.1%), *Escherichia* spp. (n = 13, 9.4%), *Citrobacter* spp. (n = 9, 6.5%), *Leclercia* spp. (n = 6, 4.3%), *Cronobacter* spp. (n = 2, 1.4%), *Raoultella* spp. (n = 1, 0.7%), and *Phytobacter* spp. (n = 1, 0.7%) (Fig. 1b). Of these *mcr-9* isolates, 77 were of human origin (55.8%), 40 of animal origin (29.0%), 5 of environment origin (3.6%) and 4 strains (2.9%) were isolated from food. The source for 12 isolates (8.7%) could not be obtained for an incomplete strain information (Fig. 1c). These data point towards a widespread dissemination of *mcr-9* among clinically important pathogens, which has serious implications on clinical therapies and public health. Active surveillance for clinical discovery of *mcr-9*-harboring pathogens is needed to provide a clue to defend against infections by superbugs with colistin resistance.

### Overall landscape of the *mcr-9*-carrying plasmids

**Figure 2:**
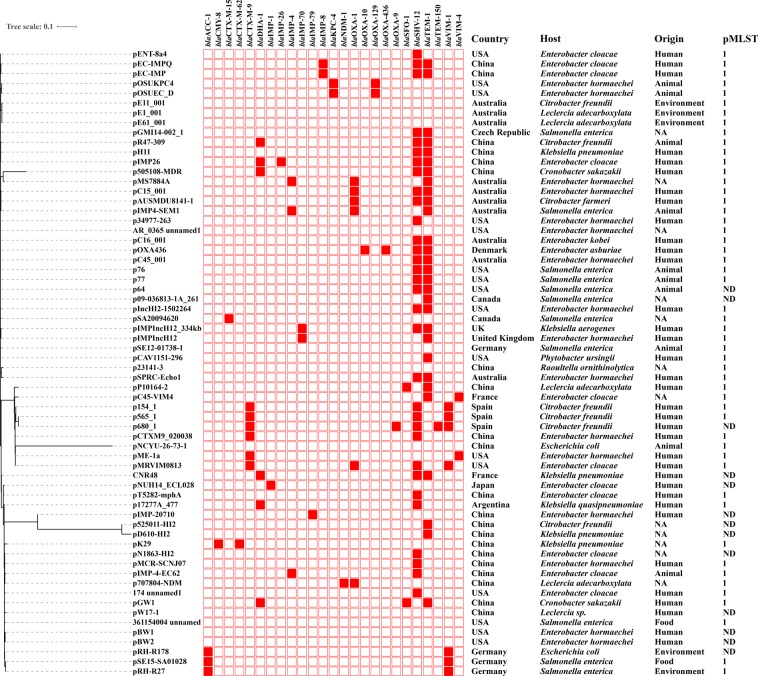
Phylogenetic analysis of IncHI2 *mcr-9*-harboring plasmids. Core-genome alignments and phylogenetic reconstruction of IncHI2 *mcr-9*-carrying plasmids were performed using Parsnp. The heatmap denotes the presence of ESBL or carbapenemase genes as determined by ABRicate. The annotation denotes (from left to right) locations, host species, isolation sources, and sequence types of plasmids. NA, not available. ND, not determined.

Seventy-one *mcr-9*-carrying plasmids with complete sequences were retrieved from the GenBank database (accessed on 12th February 2020, Table S1). Among them, IncHI2 was the dominant replicon type carrying *mcr-9*, accounting for 90.1% (64/71) of the plasmids. Of the 64 IncHI2 *mcr-9*-harboring plasmids, 60 plasmids had the IncHI2 replicon alone, 3 had IncHI2 plus IncR, and 1 had IncHI2 plus IncA/C_2_, with plasmid size varying from 222 kb to 477 kb. We found that *mcr-9* plasmids with IncHI2 replicon alone had a worldwide distribution, while plasmids containing both IncHI2 and IncR replicons had only been recovered from China, suggesting that IncHI2-IncR hybrid plasmids may represent another important vehicle in mediating the dissemination of *mcr-9* in China. Phylogenetic analysis showed that three IncHI2-IncR hybrid plasmids, pMCR-SCNJ07 (accession no. MK933279), pT5282-mphA (KY270852) and pN1863-HI2 (MF344583) were clustered with several IncHI2 plasmids (Fig. 2), implying that they may have diverged from a common ancestor and a subsequent genetic recombination event leading to the formation of hybrid plasmids. And, the IncHI2-IncA/C_2_ hybrid plasmid pGMI14-002_1 (accession no. CP028197), which was recovered from a *Salmonella enterica* isolate in Czech Republic in 2018, followed a similar pattern. From the phylogenetic tree, we also found that *mcr-9*-harboring plasmids recovered from human isolates were interspersed with plasmids from animals and the environment (Fig. 2), indicating that plasmid-borne *mcr-9* might have circulated in the entire ecosystem.

IncHI2-type plasmids have a broad host range, which represent one of the most commonly encountered plasmid groups in the *Enterobacteriaceae* family^20^. Among them, IncHI2-ST1 plasmids are known to disseminate clinically important antimicrobial resistance genes and are most frequently reported to play a critical role in the evolution of complex resistance phenotypes within disease-causing strains of *Enterobacteriaceae*^21^. Our presentation here highlighted that IncHI2-ST1 plasmids are a major vehicle carrying *mcr-9* around the world using the pMLST tool. In addition to IncHI2 plasmids, there was one IncFII (FIIY3: FIA-: FIB-) plasmid p1_045523 (accession no. CP032893) carrying *mcr-9*. And, the replicon type for 6 *mcr-9*-bearing plasmids could not be determined, with plasmid size ranging from 56 kb to 133 kb. These findings revealed that multiple types of plasmids are involved in the spread of *mcr-9*.

IncHI2 plasmids are known carriers of determinants for resistance not only to antibiotics such as β-lactams, quinolones and aminoglycosides, but also to heavy metals such as copper, silver ions, and mercuric ions^22^. Analysis of the resistance genes revealed that IncHI2 *mcr-9*-carrying plasmids contained between 0 and 5 additional ESBL or carbapenemase genes (Table S1). Among these resistance genes, *bla*_SHV-12_ was the most common one (32/71, 45.1%) that co-existed with *mcr-9*, followed by *bla*_TEM-1_ (29/71, 40.8%). In particular, 23 out of the 64 (35.9%) IncHI2 *mcr-9*-carrying plasmids harbored one of the carbapenemase genes, namely *bla*_IMP- [1, 4, 8, 26, 70, 79]_, *bla*_KPC-4_, *bla*_NDM-1_, *bla*_VIM- [1,4]_, and *bla*_OXA-436_. Co-transfer of the colistin resistance and carbapenemase genes by a single plasmid could raise the risk of dissemination of these resistance genes, which is of great concern for clinical therapies.

### Genetic characterization of plasmids carrying *mcr-9*

**Figure 3:**
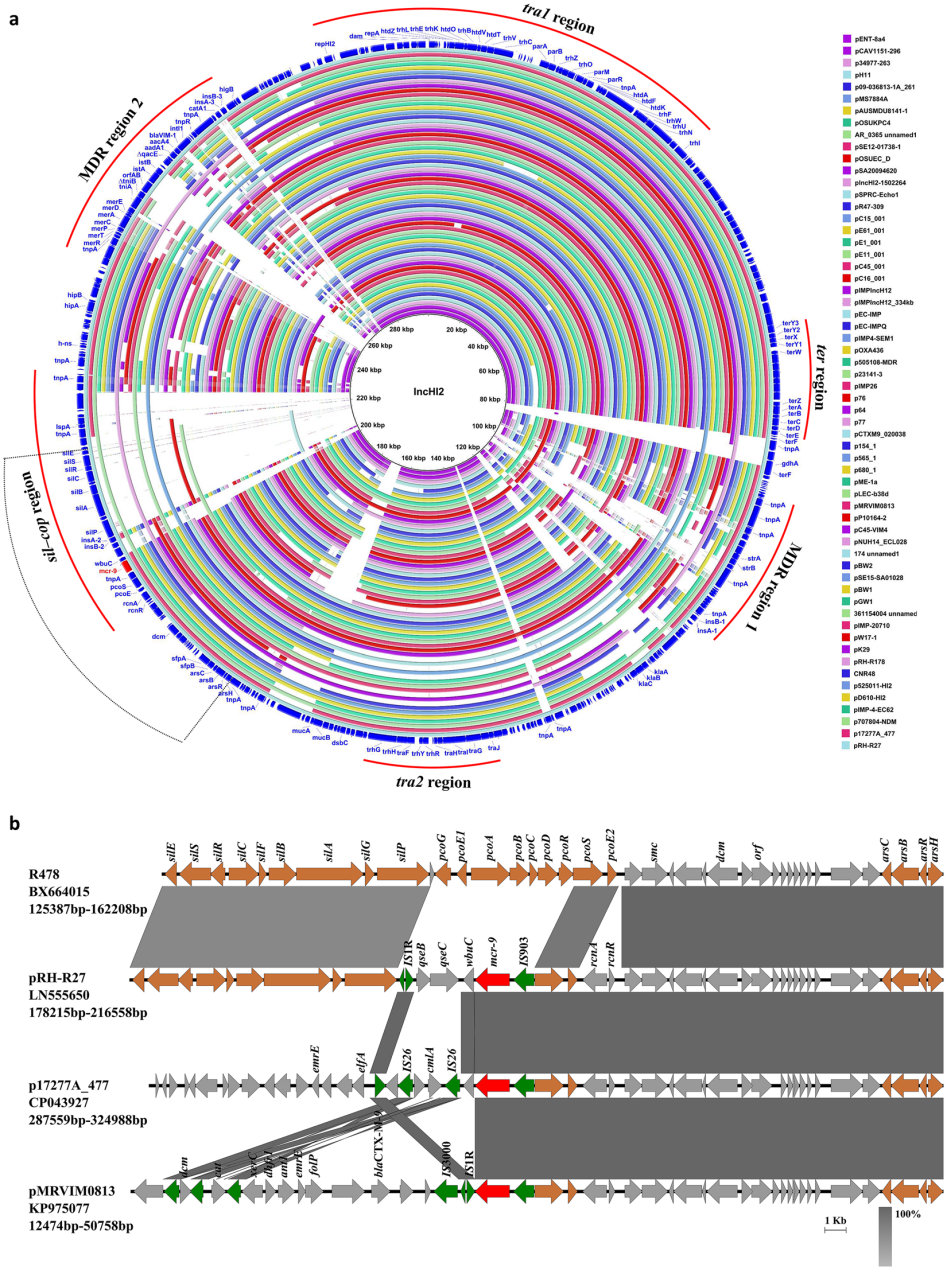
Genetic characterization of IncHI2-type *mcr-9*-harboring plasmids (hybrid plasmids are excluded). (**a)** Sequence alignment of 60 IncHI2 *mcr-9* plasmids. pRH-R27(accession no. LN555650) was used as a reference to compare with other plasmids. Gaps indicate regions that were missing in the respective plasmid compared to the reference plasmid. The outer circle with dark blue arrows denotes annotation of reference plasmid. *mcr-9* gene is highlighted by red arrows. Backbone (*tra*1 and *tra*2 region) and four accessory resistance regions (*ter* region, the *sil–cop* region, MDR region 1 and MDR region 2) are indicated by red curves. The *mcr-9*-harboring region is indicated by the dotted box. Information about the IncHI2 plasmids tested is provided in Table S1. (**b)** Linear comparison of the *mcr-9*-harboring region in pRH-R27, p17277A_477 (CP043927) and pMRVIM0813 (KP975077). The corresponding region on non-*mcr-9*-carrying plasmid R478 (BX664015, top) is shown for comparison. Grey shading denotes regions of shared homology among different plasmids ranging from 80% to100%. Colored arrows represent open reading frames, with brown, green, and red arrows representing heavy metal resistance genes, mobile elements, and the *mcr-9* gene, respectively. Other plasmid backbone genes and hypothetical genes are colored gray.

Comparative analysis was performed to obtain a comprehensive view of the genetic features of 60 IncHI2-type *mcr-9*-bearing plasmids (hybrid plasmids were excluded). Results showed that these plasmids were diverse in terms of genetic structure, except that *tra1* and *tra2* regions as well as the *ter* region were conserved among all the plasmids (Fig. 3a). *mcr-9* was located in the *sil-cop* region of IncHI2-type plasmids. In this accessory resistance region, the structure and genetic content immediately upstream of *mcr-9* were consistent among IncHI2 *mcr-9*-bearing plasmids. While, genes downstream of *mcr-9* were genetically diverse, with silver resistance determinants and *qseB/qseC* two-component regulators being absent in most plasmids (Fig. 3a,b). The *wbuC* gene, which was proposed to have transferred together with *mcr-9* as a whole fragment from *Buttiauxella spp*.^17^, was lacking downstream of *mcr-9* in some plasmids (Fig. 3a,b).

**Figure 4:**
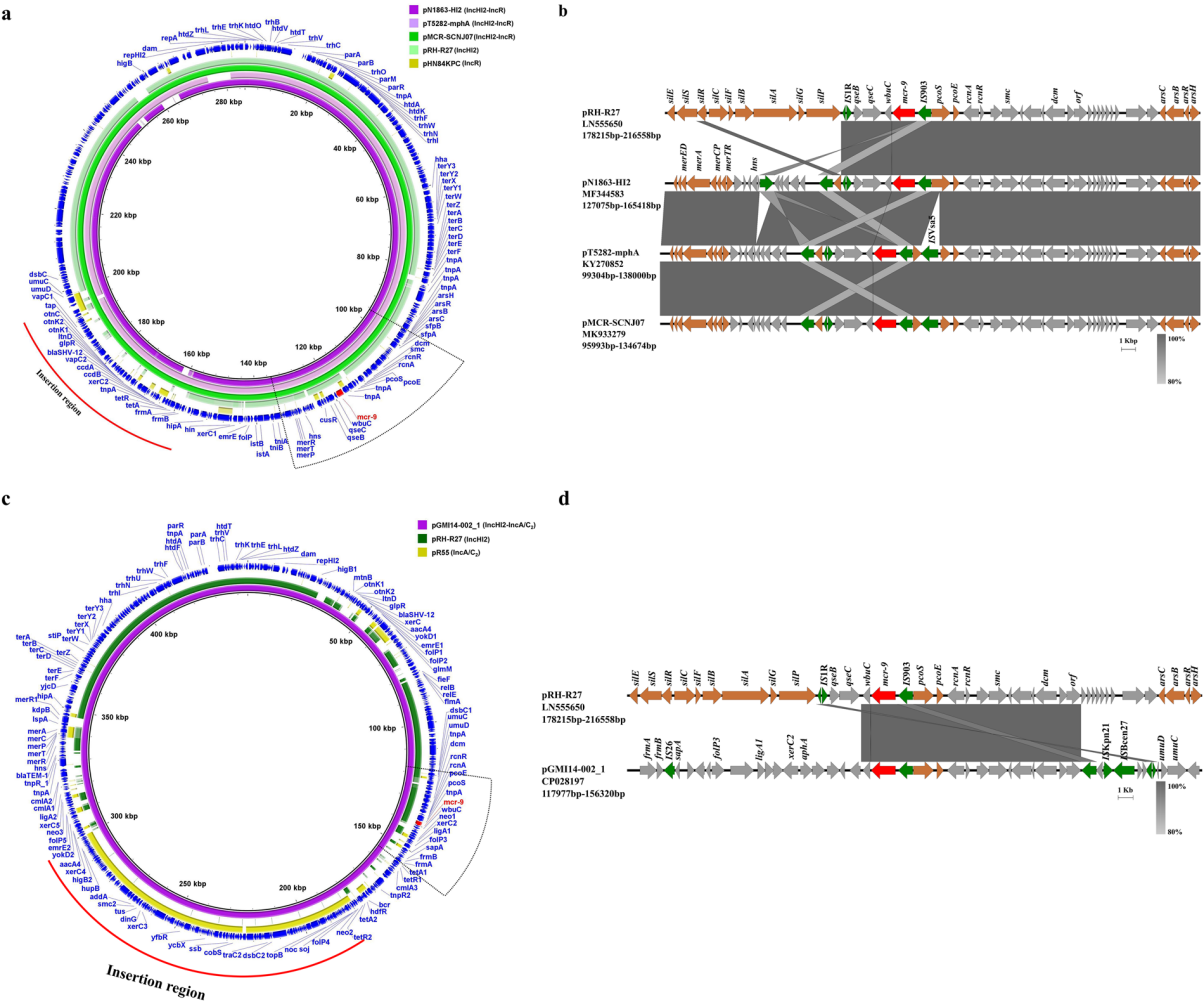
Genetic characterization of *mcr-9*-harboring hybrid plasmids. (**a)** Comparison of the IncHI2-IncR hybrid plasmids, pMCR-SCNJ07, pT5282-mphA and pN1863-HI2, with the IncHI2 plasmid pRH-R27 and IncR plasmid pHN84KPC. pN1863-HI2 was used as a reference to compare with other plasmids. (**b)** Linear comparison of the *mcr-9*-harboring region in pRH-R27, pMCR-SCNJ07, pT5282-mphA and pN1863-HI2. (**c)** Comparison of the IncHI2-IncA/C_2_ hybrid plasmid, pGMI14-002_1 with the IncHI2 plasmid pRH-R27 and IncA/C2 plasmid pR55. pGMI14-002_1 was used as a reference to compare with other plasmids. (**d)** Linear comparison of the *mcr-9*-harboring region in pRH-R27 and pGMI14-002_1. Gaps in the circular maps refer to plasmid regions that were missing in the respective plasmid compared to the reference plasmid. The outer circle with dark blue arrows denotes annotation of reference plasmid. *mcr-9* gene is highlighted by red arrows. The foreign region that was proposed to be an insertion was indicated by red curves. The *mcr-9*-harboring region is indicated by the dotted box. Grey shading in the linear maps denotes regions of shared homology among different plasmids ranging from 80% to 100%. Colored arrows represent open reading frames, with brown, green, and red arrows representing heavy metal resistance genes, mobile elements, and the *mcr-9* gene, respectively. Other plasmid genes are colored gray.

We further explored the genetic structures of the IncHI2-IncR *mcr-9-*carrying plasmids, pMCR-SCNJ07, pT5282-mphA and pN1863-HI2, by comparing them with the IncHI2 reference plasmid pRH-R27 and IncR reference plasmid pHN84KPC (accession no. KY296104). Results showed that 1) three IncHI2-IncR hybrid plasmids had similar genetic structures, 2) the hybrid plasmids showed high similarity to backbone elements of IncHI2 on a large scale (>77% query coverage, 99.99% nucleotide identity), 3) the hybrid plasmids showed low sequence homology (<9% coverage) with the IncR reference plasmid, and 4) *mcr-9* was located on the IncHI2 plasmid region (Fig. 4a). These findings suggested that multiple recombination events involving several plasmids probably contributed to the integration of foreign regions into *mcr-9*-bearing IncHI2 backbones. In a similar way, IncHI2-IncA/C_2_ hybrid plasmid pGMI14-002_1 was formed by fusion of a *mcr-9*-bearing IncHI2 plasmid and an IncA/C_2_ plasmid, like pR55 (accession no. JQ010984) (Fig. 4c). Sequence comparison of the *mcr-9*-harboring regions showed that the flanked upstream regions of *mcr-9* in IncHI2-IncR hybrid plasmids were highly similar (>93% coverage,>99.98% identity) to that of the IncHI2 reference plasmid pRH-R27, except that *pcoS* was truncated by an insertion element in pMCR-SCNJ07 and pT5282-mphA (Fig. 4b). Whereas, in comparison to pRH-R27, IncHI2-IncR plasmids lacked the *sil* resistance module downstream of *mcr-9*, with only *silR* survived, implying a relic for frequent recombination events (Fig. 4b). In the IncHI2-IncA/C_2_ plasmid, the *sil* resistance module, the *qseC-qseB* regulatory genes, and the insertion elements between them were all missing from the downstream of *mcr-9*, while the neighboring genes upstream of *mcr-9* (from *orf* to *IS*903) were identical to those in pRH-R27 (Fig. 4d).

**Figure 5:**
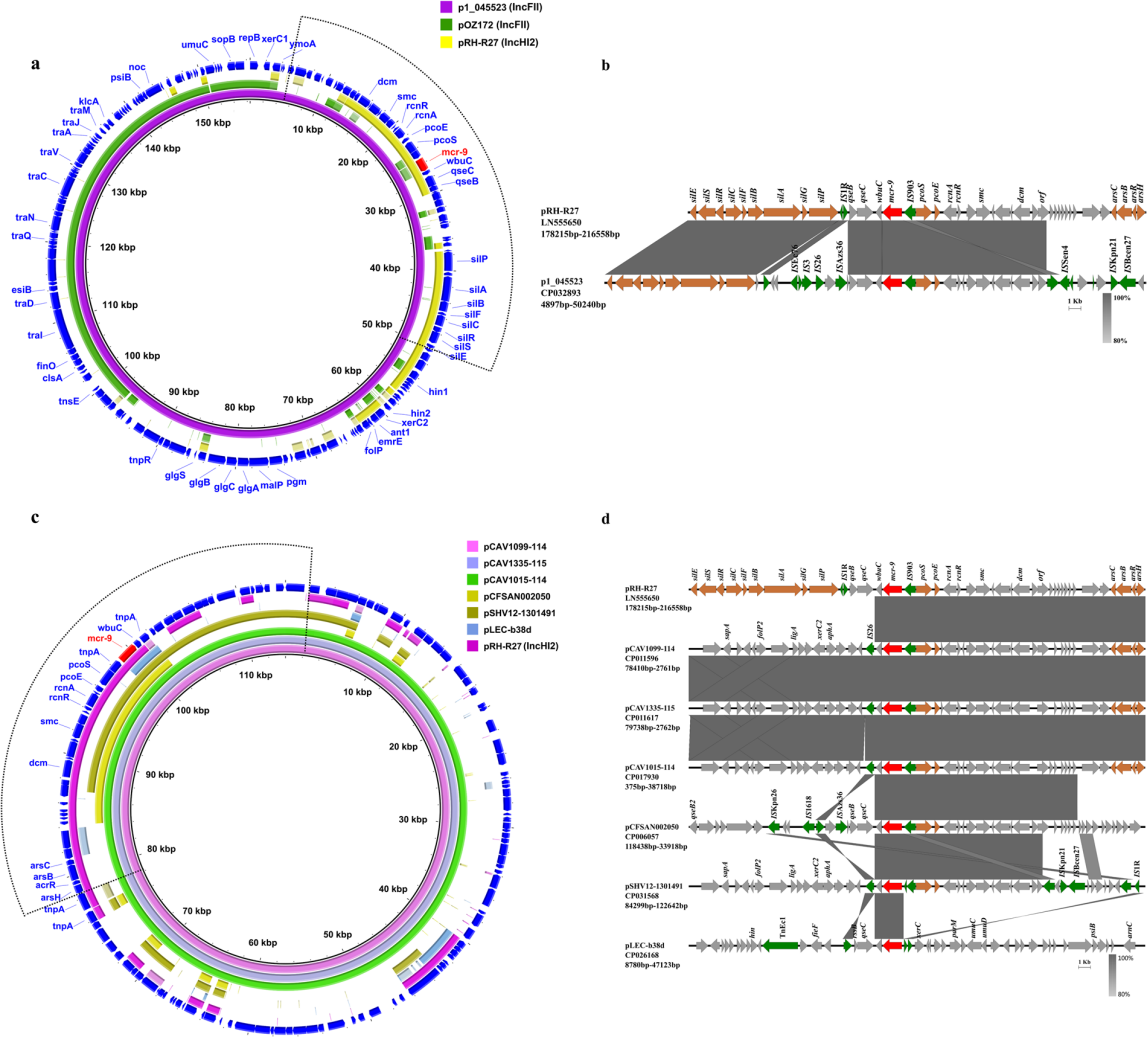
Genetic characterization of IncFII-type *mcr-9*-carrying plasmid p1_045523 and six untyped *mcr-9*-carrying plasmids. (**a)** Comparison of the p1_045523 with the IncHI2 plasmid pRH-R27 and IncFII plasmid pOZ172. p1_045523 was used as a reference to compare with other plasmids. (**b)** Linear comparison of the *mcr-9*-harboring region in pRH-R27 and p1_045523. (**c)** Comparative genome maps of pCAV1099-114 (accession no. CP011596), pCAV1335-115 (CP011617), pCAV1015-114 (CP017930), pSHV12-1301491 (CP031568), pCFSAN002050 (CP006057), pLEC-b38d (CP026168) and the IncHI2 plasmid pRH-R27. pCAV1099-114 was used as a reference to compare with other plasmids. (**d)** Linear comparison of the *mcr-9*-harboring region in six untyped *mcr-9*-carrying plasmids and pRH-R27. The outer circle with dark blue arrows denotes annotation of reference plasmid. Gaps in the circular maps refer to plasmid regions that were missing in the respective plasmid compared to the reference plasmid. *mcr-9* gene is highlighted by red arrows. The *mcr-9*-harboring region is indicated by the dotted box. Colored arrows represent open reading frames, with brown, green, and red arrows representing heavy metal resistance genes, mobile elements, and the *mcr-9* gene, respectively. The remaining genes are shown in gray. Grey shading in the linear maps denotes regions of shared homology among different plasmids ranging from 80% to 100%.

BLASTn of the IncFII *mcr-9-*carrying plasmid p1_045523 against the NCBI’s nr database identified pOZ172 (accession no. CP016763) as the highest-scoring hit, with only 50% coverage and 99.96% identity, suggesting that p1_045523 was novel in structure. Analysis of the genetic structure showed that p1_045523 contained backbone elements of the IncFII plasmid pOZ172 that encoded functions for plasmid replication and horizontal transfer, and also harbored two additional resistance gene regions (13.2–29.7 kb and 37.6–64.0 kb) displaying high similarity to the IncHI2 reference plasmid pRH-R27, with 97.80% and 99.55% identity, respectively (Fig. 5a). It was noted that *mcr-9* was exactly carried on one of the resistance regions. Comparison of the *mcr-9*-harboring regions between p1_045523 and pRH-R27 revealed that they shared regions flanking *mcr-9* (from *dcm* to *qseB*) with 100% coverage and 98.88% identity (Fig. 5b). The genetic environment of *mcr-9* in the six untyped *mcr-9*-bearing plasmids was investigated by comparing them with the IncHI2 plasmid pRH-R27. Results showed that an *mcr-9-*harboring region (from *dcm* to *wbuC*) was consistent among them, except in the plasmid pLEC-b38d, in which only the *mcr-9-wbuC* module retained (Fig. 5c,d). Our findings suggested that these untyped plasmids (excluding pLEC-b38d) were most likely to have acquired the *mcr-9* gene from IncHI2-type plasmids by multiple recombination events, and pLEC-b38d might have evolved earlier with the possibility of different patterns for the acquisition and dissemination of *mcr-9*. In addition, *mcr-9* was also found to have a chromosomal location in 6 *Enterobacteriaceae* strains (Fig. S1), with identical core structure of *mcr-9* cassettes to the IncHI2 plasmid pRH-R27, implying a relic of recombination/integration between the plasmid and chromosome.

Analysis on the plasmid reservoirs of *mcr-9* gene highlighted a leading role of IncHI2-type plasmid in the dissemination of *mcr-9*. Searches for IncHI2 *mcr*-harboring plasmids deposited in GenBank suggested that IncHI2 plasmids also served as an important vehicle in carrying *mcr-1* and *mcr-3*. Unlike the scenario seen with the *mcr-1* or *mcr-3*, which was inserted into different genetic loci in IncHI2 plasmids, *mcr-9* was consistently located in the *sil-cop* region (Figs. S2, 3a). These results indicated that *mcr-9* was inactive during dynamic gene transposition events, suggesting that transposon elements of *mcr-9* could be lost soon after the mobilization of *mcr-9* onto the IncHI2 backbone.

**Figure 6:**
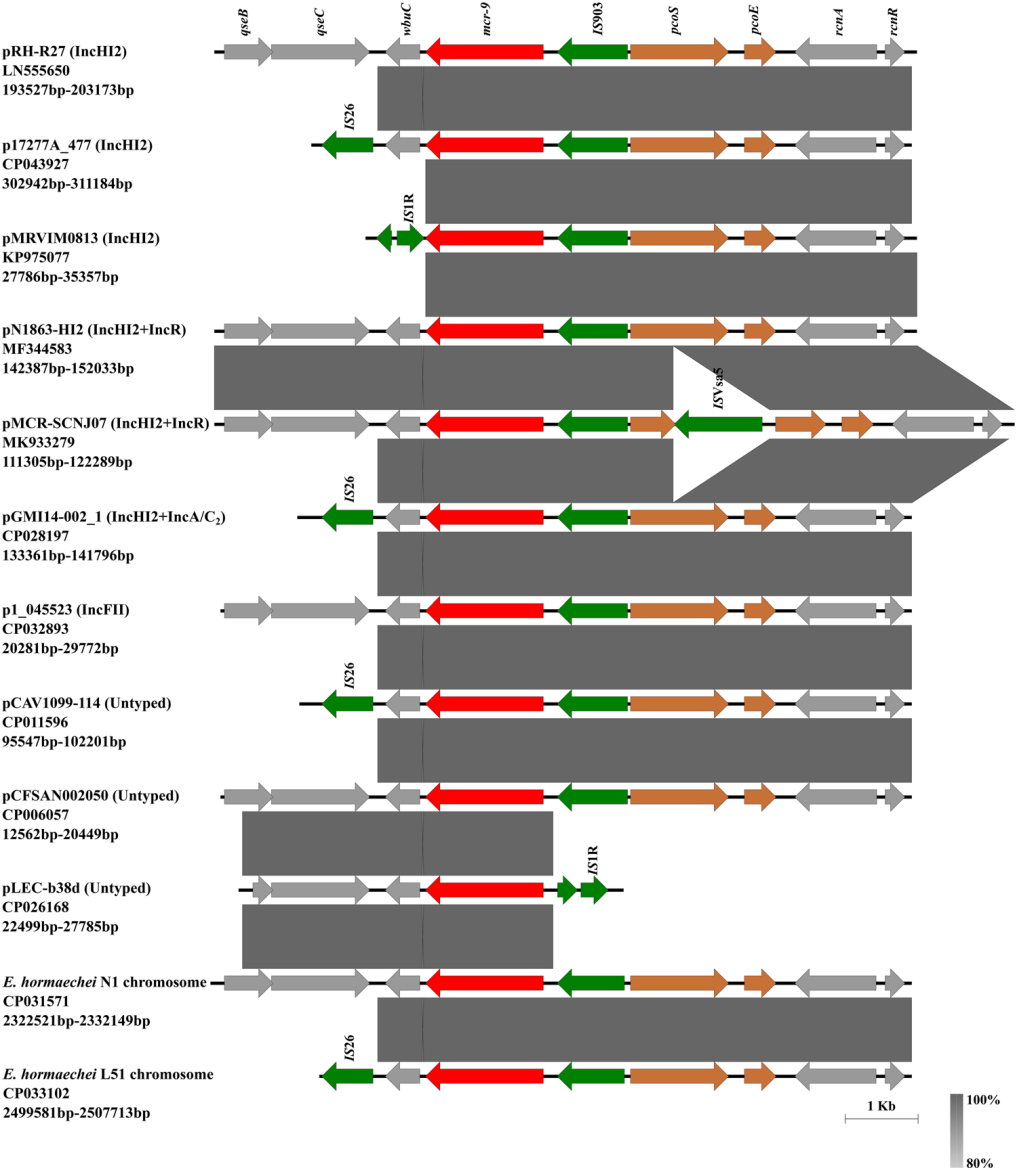
A summary of representative genetic contexts of *mcr-9* from different types of plasmid and chromosomes available to date. The genetic contexts of *mcr-9* were differed by the truncation status of neighboring genes (from *rcnR* to *qseB*) encompassing *mcr-9*. The ‘*rcnR-rcnA-pcoE-pcoS-IS*903*-mcr-9*-*wbuC*’ structure was revealed as the core structure of *mcr-9* cassettes by comparison analysis. Colored arrows represent open reading frames, with brown, green, and red arrows representing heavy metal resistance genes, mobile elements, and the *mcr-9* gene, respectively. The remaining genes are shown in gray. Grey shading denotes genetic regions that exhibit sequence homology among different segments.

To further characterize the genetic contexts of *mcr-9*, representative *mcr-9-*haboring regions from different types of plasmids and chromosomes were subjected to comparison analysis. *mcr-9* was found in a variety of genetic contexts, which were differed by the truncation status of neighboring genes (from *rcnR* to *qseB*) encompassing *mcr-9*. Results also revealed that the core structure of all known *mcr-9* cassettes was *rcnR-rcnA-pcoE-pcoS-IS*903*-mcr-9*-*wbuC* (Fig. 6), and it was identical in most (86.0%, 61/71) of the study plasmids, indicating that these core elements encompassing *mcr-9* are most likely to be required for the evolution of *mcr-9* cassettes. We failed to identify potential transposon elements (intact or truncated) mediating the transfer of *mcr-9*, posing the high possibility that conjugation and recombination events of *mcr-9*-carrying plasmids are mainly responsible for the spread of *mcr-9*. The lacking of the downstream two-component regulatory genes *qseC* and *qseB*, which were proposed to be involved in the induction of colistin resistance mediated by *mcr-9*, was commonly observed among the *mcr-9*-haboring plasmids (Figs. 3–6). More research should be performed to confirm the essential role of *qseC*-*qseB* module or the involvement of other genes in *mcr-9* induction.

## Conclusion

In conclusion, we assembled the data set to date of *mcr-9*-positive sequenced plasmids and chromosomes through an exhaustive search of publicly available databases. This study allowed us to obtain a clear picture of the global distribution of *mcr-9*-harboring isolates, which covered 21 countries across six continents and were distributed in at least 9 *Enterobacteriaceae* species of diverse origins. The co-transfer of *mcr-9* and carbapenemase genes in clinically important pathogens posed a high risk to the public health and clinical treatment. IncHI2-ST1 plasmid was found to be the predominant replicon type carrying *mcr-9*, implying that IncHI2-ST1 plasmids may be a major vehicle in mediating the dissemination *mcr-9*. Besides, our results extended significantly our understanding of genetic features of different types of *mcr-9*-bearing plasmids, and highlighted that IncHI2-type plasmids serve as a critical reservoir of *mcr-9*. In addition, the highly similarity of *mcr-9* cassette in different plasmids posed the possibility that the dissemination of *mcr-9* across bacterial populations was mainly mediated by conjugation and recombination events of *mcr-9*-carrying plasmids. A limitation of this study lies in that the phenotypes of the *mcr-9*-harboring strains are not available, which makes it difficult to correlate phenotypic with genotypic features. Whole-genome sequencing based surveillance and epidemiological studies are needed to achieve better insights into the genetic features of *mcr-9*-positive strains, which might eventually allow us to develop effective strategies to manage their spread.

## Materials and Methods

### Retrieval of genomic data and literature search

To obtain the *mcr-9*-harboring genome sequences, a nucleotide BLAST with standard options was performed in the NCBI GenBank database with the nucleotide base sequence of *mcr-9* as a query (GenBank accession no. MK791138). Models and uncultured environmental samples were excluded. Complete plasmids and chromosomes containing at least one contig with a full-length hit to *mcr-9* with 100% query coverage and at least 99% identity were selected and exported. Information about geographic distribution and isolation source of the *mcr-9*-positive strains were investigated. Additionally, relevant papers that published on *mcr-9* were identified in PubMed and Google Scholar using the query ‘*mcr-9* AND colistin’, and non-repetitive information of the geographic and isolation source of *mcr-9* strains were included in the statistics.

### Phylogenetic analysis

The complete sequences of all available *mcr-9*-carrying plasmids as of 12th February 2020 were retrieved from the GenBank. The plasmid replicon type and MLST were determined using the Plasmidfinder (https://cge.cbs.dtu.dk/services/PlasmidFinder/) and pMLST (https://cge.cbs.dtu.dk/services/pMLST/). ResFinder (http://genomicepidemiology.org/) was employed for the identification of antimicrobial resistance genes. All the IncHI2 *mcr-9*-carrying plasmids were used for phylogenetic analysis using Parsnp in the Harvest package^23^, and the phylogenetic tree was visualized by iTOL (https://itol.embl.de/).

### Comparative analysis of *mcr-9*-harboring genome sequences

The annotations of *mcr-9*-carrying plasmids were performed using Prokka^24^ combined with BLASTp/BLASTn searches against the NCBI database. Alignments among highly homologous complete plasmid sequences were performed using BLAST and visualized with the BRIG tool^25^. Sequence alignments among *mcr-9*-carrying plasmids or chromosomes were performed using BLAST and visualized with Easyfig v 2.2.3^26^.

## Supplementary information

Supplementary information.
