# Supplementary material for: Characterization of the global distribution and diversified plasmid reservoirs of the colistin resistance gene mcr-9
Source: Sci Rep. 2020 May 15;10:8113. doi: 10.1038/s41598-020-65106-w (PMC7229202; doi:10.1038/s41598-020-65106-w)
Supplement: Supplementary file 1 — Supplementary information. [file 41598_2020_65106_MOESM1_ESM.docx]

Characterization of the global distribution and diversified plasmid reservoirs of the colistin resistance gene *mcr-9*

**Ying Li^1^, Xiaoyi Dai^2^, Jing Zeng^2^, Yan Gao^1^, Zhikun Zhang^2^, Luhua Zhang^2*^**

1 Department of Immunology, School of Basic Medical Sciences, Southwest Medical University, Luzhou, Sichuan, China;

2 Department of Pathogenic Biology, School of Basic Medical Sciences, Southwest Medical University, Luzhou, Sichuan, China.

*Correspondence: zhluhua@swmu.edu.cn; Tel.: +86–0830–3160073 (L.Z)

**Table S1 Detailed information of *mcr-9*-carrying plasmids（last accessed 12 February, 2020）**

| Accession no. | plasmid | Host strain | Country | Year | Origin | Plasmid replicon | Carbapenemase  /ESBL gene |
| --- | --- | --- | --- | --- | --- | --- | --- |
| CP038659 | p680_1 | *Citrobacter freundii* | Spain | 2014 | Human | IncHI2 | *bla*_CTX-M-9_, *bla*_SHV-12_, *bla*_VIM-1_, *bla*_OXA-9_, *bla*_TEM-150_ |
| CP038657 | p565_1 | *Citrobacter freundii* | Spain | 2014 | Human | IncHI2 | *bla*_CTX-M-9_, *bla*_SHV-12_, *bla*_VIM-1_ |
| CP038654 | p154_1 | *Citrobacter freundii* | Spain | 2014 | Human | IncHI2 | *bla*_CTX-M-9_, *bla*_SHV-12_, *bla*_VIM-1_ |
| CP041734 | pME-1a | *Enterobacter hormaechei* | USA | 2018 | Human | IncHI2 | *bla*_CTX-M-9_, *bla*_VIM-4_ |
| CP031724 | pCTXM9_020038 | *Enterobacter hormaechei* | China | 2016 | Human | IncHI2 | *bla*_CTX-M-9_, *bla*_SHV-12_, |
| LT991958 | pC45-VIM4 | *Enterobacter cloacae* | France | 2014 | NA | IncHI2 | *bla*_TEM-1_, *bla*_VIM-4_ |
| KX710093 | pP10164-2 | *Leclercia adecarboxylata* | China | 2012 | Human | IncHI2 | *bla*_SFO-1_, *bla*_TEM-1_ |
| KP975077 | pMRVIM0813 | *Enterobacter cloacae* | USA | 2015 | Human | IncHI2 | *bla*_CTX-M-9_, *bla*_SHV-12_, *bla*_VIM-1_, *bla*_OXA-1_ |
| CP027112 | pBW1 | *Enterobacter hormaechei* | USA | 2014 | Human | IncHI2 |  |
| CP023570 | pBW2 | *Enterobacter hormaechei* | USA | 2013 | Human | IncHI2 |  |
| CP042552 | pC45_001 | *Enterobacter hormaechei* | Australia | 2013 | Human | IncHI2 | *bla*_SHV-12_, *bla*_TEM-1_ |
| CP043767 | pIMPIncH12 | *Enterobacter hormaechei* | United Kingdom | 2019 | Human | IncHI2 | *bla*_IMP-70_, *bla*_TEM-1_ |
| CP042525 | pE11_001 | *Citrobacter freundii* | Australia | 2012 | Environment | IncHI2 |  |
| CP042579 | pC16_001 | *Enterobacter kobei* | Australia | 2009 | Human | IncHI2 | *bla*_SHV-12_, *bla*_TEM-1_ |
| CP042494 | pE61_001 | *Leclercia adecarboxylata* | Australia | 2014 | Environment | IncHI2 |  |
| CP042489 | pC15_001 | *Enterobacter hormaechei* | Australia | 2009 | Human | IncHI2 | *bla*_SHV-12_, *bla*_TEM-1_, *bla*_OXA-1_ |
| CP042506 | pE1_001 | *Leclercia adecarboxylata* | Australia | 2012 | Environment | IncHI2 |  |
| CP044215 | pIMPIncH12_334kb | *Klebsiella aerogenes* | United Kingdom | 2019 | Human | IncHI2 | *bla*_IMP-70_, *bla*_SHV-12_, *bla*_TEM-1_ |
| CP043927 | p17277A_477 | *Klebsiella quasipneumoniae* | Argentina | 2014 | Human | IncHI2 | *bla*_SHV-12_, *bla*_DHA-1_ |
| CP040696 | pR47-309 | *Citrobacter freundii* | China | 2017 | Animal | IncHI2 | *bla*_DHA-1_, *bla*_SHV-12_, *bla*_TEM-1_ |
| CP020529 | 174 unnamed1 | *Enterobacter cloacae* | USA | 2015 | Human | IncHI2 | *bla*_SHV-12_ |
| MH909331 | p707804-NDM | *Leclercia adecarboxylata* | China | 2018 | NA | IncHI2 | *bla*_NDM-1_, *bla*_OXA-1_ |
| MK191844 | p77 | *Salmonella enterica* | USA | 2018 | Animal | IncHI2 | *bla*_SHV-12_, *bla*_TEM-1_ |
| MK191841 | p64 | *Salmonella enterica* | USA | 2018 | Animal | IncHI2 | *bla*_SHV-12_, *bla*_TEM-1_ |
| MK191835 | p76 | *Salmonella enterica* | USA | 2018 | Animal | IncHI2 | *bla*_SHV-12_, *bla*_TEM-1_ |
| CP028975 | pGW1 | *Cronobacter sakazakii* | China | 2015 | Human | IncHI2 | *bla*_DHA-1_, *bla*_TEM-1_, *bla*_SFO-1_ |
| MH829594 | pIMP-4-EC62 | *Enterobacter cloacae* | China | 2018 | Animal | IncHI2 | *bla*_SHV-12_, *bla*_IMP-4_ |
| CP032842 | pSPRC-Echo1 | *Enterobacter hormaechei* | Australia | 2007 | Human | IncHI2 | *bla*_SHV-12_, *bla*_TEM-1_ |
| MH399264 | pIMP26 | *Enterobacter cloacae* | China | 2013 | Human | IncHI2 | *bla*_SHV-12_, *bla*_TEM-1_, *bla*_IMP-26_, *bla*_DHA-1_ |
| CP031575 | pIncHI2-1502264 | *Enterobacter hormaechei* | USA | 2015 | Human | IncHI2 | *bla*_SHV-12_, *bla*_TEM-1_ |
| CP029248 | pOSUEC_D | *Enterobacter hormaechei* | USA | 2016 | Animal | IncHI2 | *bla*_KPC-4_, *bla*_OXA-129_ |
| CP031102 | pW17-1 | *Leclercia sp.* | China | 2018 | Human | IncHI2 |  |
| CP030186 | pSA20094620 | *Salmonella enterica* | Canada | 2018 | NA | IncHI2 | *bla*_CTX-M-15_ |
| LT994835 | CNR48 | *Klebsiella pneumoniae* | France | 2018 | Human | IncHI2 | *bla*_SHV-12_, *bla*_TEM-1_, *bla*_DHA-1_ |
| CP030080 | pIMP-20710 | *Enterobacter hormaechei* | China | 2011 | Human | IncHI2 | *bla*_IMP-79_ |
| CP024910 | pOSUKPC4 | *Enterobacter hormaechei* | USA | 2016 | Animal | IncHI2 | *bla*_KPC-4_, *bla*_OXA-129_ |
| CP029037 | 361154004 unnamed | *Salmonella enterica* | USA | 2009 | Food | IncHI2 |  |
| CP027678 | pSE12-01738-1 | *Salmonella enterica* | Germany | 2012 | Animal | IncHI2 |  |
| MG288680 | pD610-HI2 | *Klebsiella pneumoniae* | China | 2017 | NA | IncHI2 | *bla*_TEM-1_ |
| CP026661 | pSE15-SA01028 | *Salmonella enterica* | Germany | 2015 | Food | IncHI2 | *bla*_ACC-1_, *bla*_VIM-1_ |
| CP027144 | AR_0365 unnamed1 | *Enterobacter hormaechei* | USA | 2018 | NA | IncHI2 |  |
| MF344582 | p525011-HI2 | *Citrobacter freundii* | China | 2017 | NA | IncHI2 | *bla*_TEM-1_ |
| MF788071 | p23141-3 | *Raoultella ornithinolytica* | China | 2017 | NA | IncHI2 |  |
| CP022696 | pAUSMDU8141-1 | *Citrobacter farmeri* | Australia | 2015 | Human | IncHI2 | *bla*_SHV-12_, *bla*_TEM-1_, *bla*_OXA-1_ |
| KY978628 | p505108-MDR | *Cronobacter sakazakii* | China | 2016 | Human | IncHI2 | *bla*_SHV-12_, *bla*_TEM-1_, *bla*_DHA-1_ |
| CP022533 | pMS7884A | *Enterobacter hormaechei* | Australia | 2017 | NA | IncHI2 | *bla*_IMP-4_, *bla*_OXA-1_ *bla*_TEM-1_, |
| KY863418 | pOXA436 | *Enterobacter asburiae* | Denmark | 2014 | Human | IncHI2 | *bla*_SHV-12_, *bla*_TEM-1_, *bla*_OXA-10_, *bla*_OXA-436_ |
| KX810825 | pIMP4-SEM1 | *Salmonella enterica* | Australia | 2016 | Animal | IncHI2 | *bla*_IMP-4_, *bla*_OXA-1_, *bla*_TEM-1_, |
| CP016526 | p09-036813-1A_261 | *Salmonella enterica* | Canada | 2016 | NA | IncHI2 | *bla*_TEM-1_ |
| CP013215 | pH11 | *Klebsiella pneumoniae* | China | 2010 | Human | IncHI2 | *bla*_SHV-12_, *bla*_TEM-1_ |
| LN555650 | pRH-R27 | *Salmonella enterica* | Germany | 2014 | Environment | IncHI2 | *bla*_ACC-1_, *bla*_VIM-1_ |
| CP012170 | p34977-263 | *Enterobacter hormaechei* | USA | 2009 | Human | IncHI2 | *bla*_SHV-12_ |
| CP011601 | pCAV1151-296 | *Phytobacter ursingii* | USA | 2009 | Human | IncHI2 | *bla*_TEM-1_ |
| CP008899 | pENT-8a4 | *Enterobacter cloacae* | USA | 2011 | Human | IncHI2 | *bla*_SHV-12_ |
| EU855788 | pEC-IMPQ | *Enterobacter cloacae* | China | 2008 | Human | IncHI2 | *bla*_IMP-8_, *bla*_SHV-12_, *bla*_TEM-1_ |
| EU855787 | pEC-IMP | *Enterobacter cloacae* | China | 2008 | Human | IncHI2 | *bla*_IMP-8_, *bla*_SHV-12_, *bla*_TEM-1_ |
| EF382672 | pK29 | *Klebsiella pneumoniae* | China | 2007 | NA | IncHI2 | *bla*_CMY-8_, *bla*_CTX-M-62_ |
| CP042616 | pNCYU-26-73-1 | *Escherichia coli* | China | 2017 | Animal | IncHI2 |  |
| HG530658 | pRH-R178 | *Escherichia coli* | Germany | 2013 | Environment | IncHI2 | *bla*_ACC-1_, *bla*_VIM-1_ |
| AP019384 | pNUH14_ECL028 | *Enterobacter cloacae* | Japan | 2014 | Human | IncHI2 | *bla*_IMP-1_ |
| CP032893 | p1_045523 | *Enterobacter kobei* | China | 2017 | Human | IncFII |  |
| CP028197 | pGMI14-002_1 | *Salmonella enterica* | Czech Republic | 2018 | NA | IncA/C2  IncHI2 | *bla*_SHV-12_, *bla*_TEM-1_ |
| MF344583 | pN1863-HI2 | *Enterobacter cloacae* | China | 2017 | NA | IncHI2  IncR | *bla*_SHV-12_ |
| KY270852 | pT5282-mphA | *Enterobacter cloacae* | China | 2016 | Human | IncHI2  IncR | *bla*_SHV-12_ |
| MK933279 | pMCR-SCNJ07 | *Enterobacter hormaechei* | China | 2018 | Human | IncHI2  IncR | *bla*_SHV-12_ |
| CP031568 | pSHV12-1301491 | *Enterobacter hormaechei* | USA | 2013 | Human | NA | *bla*_SHV-12_ |
| CP026168 | pLEC-b38d | *Leclercia sp.* | USA | 2016 | NA | NA |  |
| CP011617 | pCAV1335-115 | *Klebsiella oxytoca* | USA | 2010 | Human | NA |  |
| CP011596 | pCAV1099-114 | *Klebsiella oxytoca* | USA | 2009 | Human | NA |  |
| CP006057 | pCFSAN002050 | *Salmonella enterica* | USA | 2012 | Food | NA |  |
| CP017930 | pCAV1015-114 | *Klebsiella oxytoca* | USA | 2007 | Human | NA |  |

NA: not available;


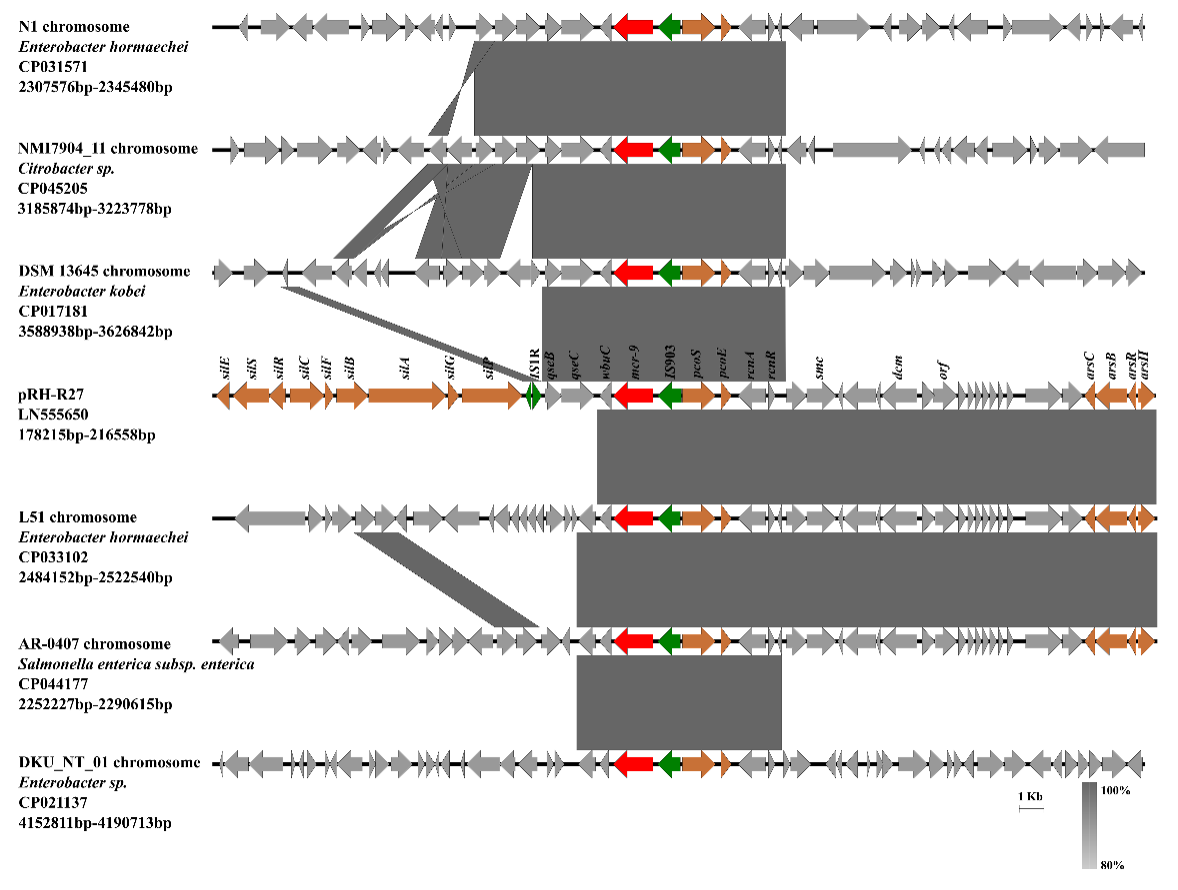


Figure S1. Characterization of the genetic contexts of *mcr-9* on the chromosomes. The corresponding region on IncHI2 *mcr-9*-carrying plasmid pRH-R27 is shown for comparison. Colored arrows represent open reading frames, with brown, green, and red arrows representing heavy metal resistance genes, mobile elements, and the *mcr-9* gene, respectively. The remaining genes are shown in gray. Grey shading denotes genetic regions that exhibit sequence homology among different segments.


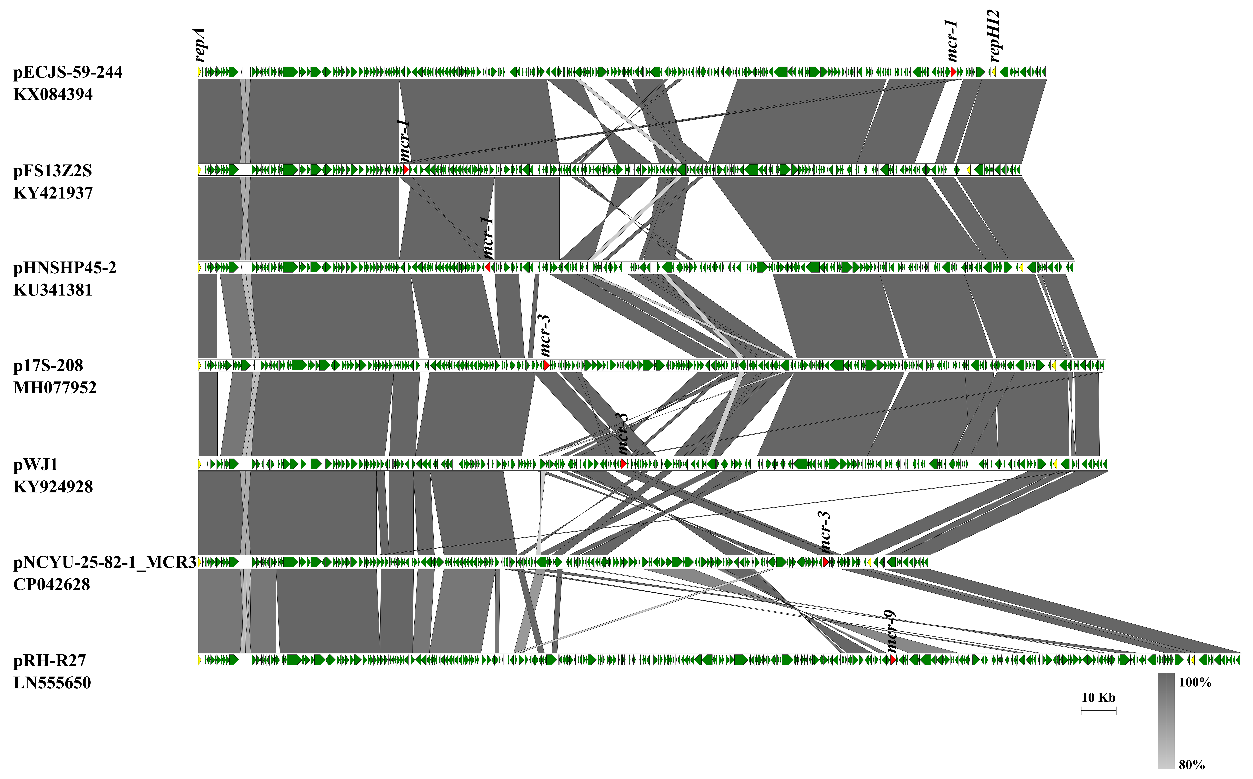


**Figure S2.** Linear comparison of IncHI2-type plasmids carrying *mcr-1, mcr-3* or *mcr-9*. The arrows represent the position and transcriptional direction of the ORFs. *mcr* genes are highlighted by red arrows. Replicons of the plasmids are shown in yellow. The remaining genes are shown in green. Grey shading in the linear maps denotes regions of shared homology among different plasmids ranging from 80% to 100%.
